# Performance on functional capacity tests and level of physical activity in women with metabolic syndrome

**DOI:** 10.1186/1758-5996-7-S1-A233

**Published:** 2015-11-11

**Authors:** Caroline Hellen Rampazzo Alves, Shirley Aparecida Fabris de Souza, Nathália Caroline Valentini de Azevedo, Décio Sabbatini Barbosa, Danielle Venturini, Alessandra M Okino

**Affiliations:** 1 Universidade Estadual de Londrina, Londrina, Brazil

## Background

Metabolic Syndrome (MetS) is a condition associated an increased risk for type 2 diabetes and heart disease as hypertension, high triglyceride levels, low HDL cholesterol levels, and above-normal blood glucose levels.

## Objective

To assess the body composition and the responses of functional capacity influence the level of physical activity in women with MetS.

## Materials and methods

This cross-sectional study consisted in 59 women, with age range from 30 to 55 yrs., were divided into two groups: I.MetS (patients with Metabolic Syndrome, n=36) and II.Control (normal subjects, n=23). The diagnosis of MetS was done through a clinical and laboratory evaluation according to the National Cholesterol Education Program-Adult Treatment Panel III (NCEP-ATP III). To assess the physical activity level was applied the International Physical Activity Questionnaire [IPAQ] and performed two functional tests: Step Test (ST) of 2 min and Sit-To-Stand Test and chair (STS) of 30 second, assessing the aerobic capacity exercise, functional status and strength of the lower limbs. Statistical analysis was performed by the Statistical Package for Social Sciences (SPSS-2.0). Statistical significance was accepted when p<0.05. The Chi-square Test for categorical data analysis, numeric data Spearman correlation was performed.

## Results

There was no significant difference between groups I and II on the tests and in the questionnaire. In group I, 41.7% are inactive and Group II, 56.5% are minimally active (p=0,360). On the ST was found an average performance of 48.5 repetitions (p=0.597), and 12 repeats in the STS (p=0.267) in group I. Group II 50 repetitions in ST and 13 repetitions in the STS. The Results of IPAQ questionnaire in Group I was 1732 MET and Group II 2500 MET (p=0.744). There was a positive correlation between STS and TD (rs=0.56/p=0.000) and between the TSL chair and age (rs=0.684/p=0.000).

## Conclusion

The proportion of sedentary time was strongly related to metabolic risk, independent of physical activity. The physical activity should be encouraged for both healthy people and especially for those with MetS, helping to reduce risk factors.

**Figure 1:**
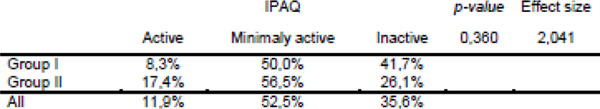
Values in the level of International Physical Activity Question (IPAW) in groups I and II.

**Figure 2:**
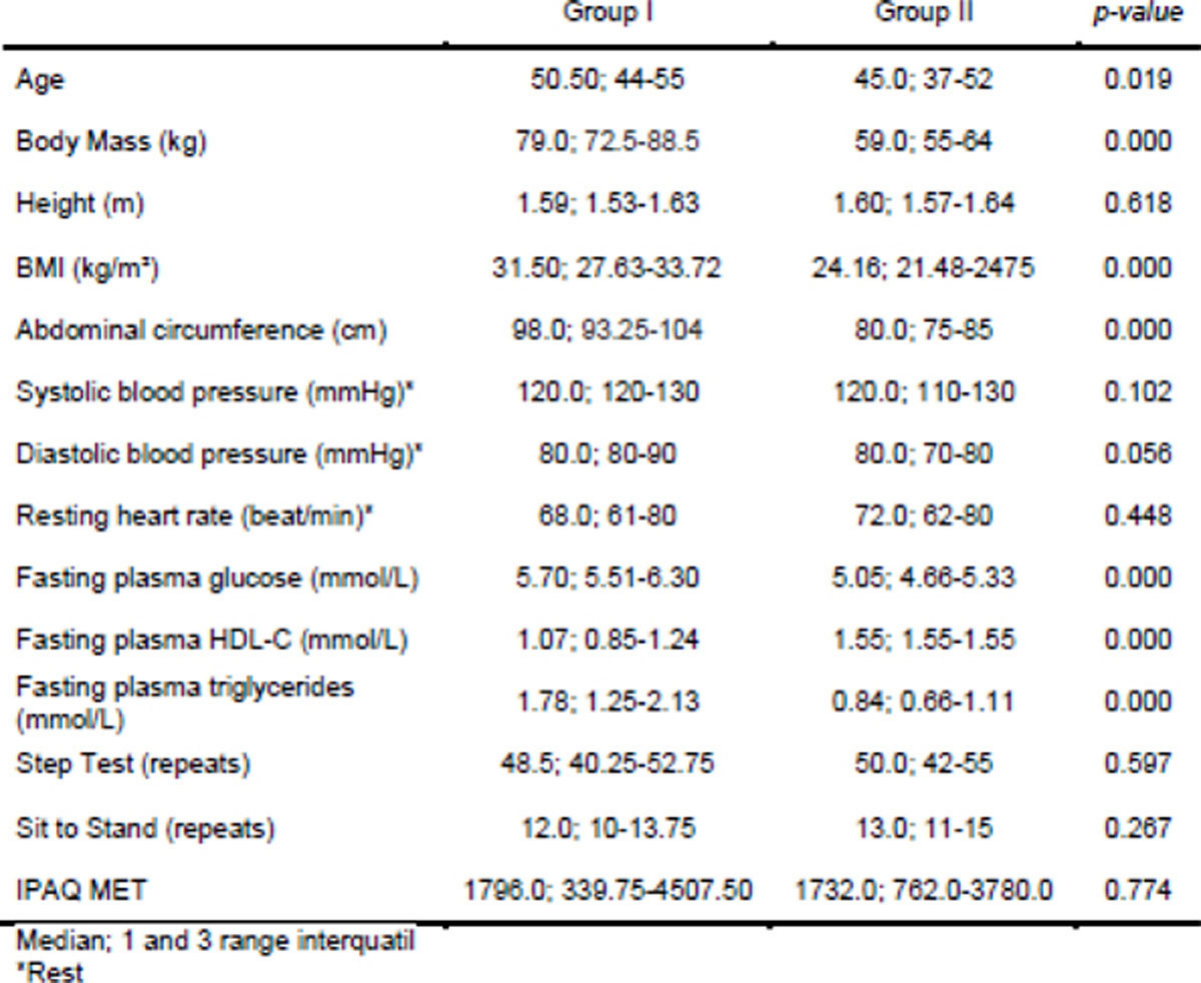
Comparison of descriptive characteristics between Groups I and II with MetS and without MetS.

